# Impact of sports vision training on visuomotor skills and shooting performance in elite skeet shooters

**DOI:** 10.3389/fnhum.2024.1476649

**Published:** 2024-11-13

**Authors:** Yuqiang Guo, Tinggang Yuan, Jian Peng, Liwei Deng, Chao Chen

**Affiliations:** ^1^Science Research Center of Sports Training, China Institute of Sport Science, Beijing, China; ^2^School of Physical Education, Shanghai University of Sport, Shanghai, China; ^3^School of Physical Education, Hunan University of Science and Technology, Changsha, China; ^4^School of Physical Education, Liaocheng University, Liaocheng, China; ^5^College of Physical Education, Dalian University, Dalian, China

**Keywords:** sports vision testing, perceptual and cognitive performance, reaction time, shotgun kinematics analysis, Senaptec Sensory Station

## Abstract

**Introduction:**

Vision serves as a critical channel for athletes to acquire information during competitions and constitutes a vital component of their competitive ability. Through scientifically designed sports visual training, specific visual skills can be enhanced, thereby assisting athletes in achieving optimal performance in competitive settings. This study aim to explore the visuomotor abilities and shooting performance of skeet shooters through Sports Vision Training (SVT).

**Methods:**

Twenty elite skeet shooters were recruited and randomly assigned to an experimental group (EXP, *n* = 10) and a control group (CON, *n* = 10). The EXP underwent 6-week of SVT on Senaptec Seneory Station, twice a week, while the CON completed an equivalent workload of target-tracking training.

**Result:**

Visuomotor skills testing showed significant improvements in Near- Far Quickness, Perception Span, and Eye-hand Coordination in the EXP (*p* < 0.05), with no changes in the CON. Comparative post-test results between the two groups showed significant differences in N/F Q, Target Capture, Perception Span, Eye-hand Coordination, and Go/ No Go (*p* < 0.05). In shooting performance indicators, EXP shooters showed a highly significant improvement in hit accuracy (*p* < 0.01), with a similar difference compared to the CON. Additionally, they exhibited a highly significant improvement in shotgun-mounting reaction time (*p* < 0.01). Kinematic indicators of shotgun movement during the firing process for shot 2 showed significant differences in peak velocity (*p* < 0.01), X-axis (*p* = 0.033) and Y-axis (*p* = 0.001) displacement.

**Conclusion:**

SVT can enhance visuomotor abilities in skeet shooters and has a positive impact on their shooting technique. This is primarily manifested in shorter shotgun-mounting reaction time and improved efficiency in action at shot 2, effectively improving their shooting accuracy.

## 1 Introduction

Sports vision refers to the observational and feedback capabilities utilized during athletic activities, encompassing components such as visual serach, visual attention and the ability to select the relevant informantion. These aspects play a crucial role in how athletes interpret their environment and make informed decisions (Appelbaum and Erickson, 2018; Buscemi et al., 2024; Millard et al., 2023). For instance, research by Stein et al. (2024) emphasizes the importance of eye and hand movements in visual search strategies, while Kassem et al. (2024) examines the eye movements in relation to decision-making and game knowledge, and the final findings can serve as a tool for talent indentification processes. Over 80% of sensory information that athletes acquire during competition comes from the visual system (Joseph et al., 2020). Mastering sports techniques involves not only training the muscular system but also enhancing brain functions related to vision (Ellison et al., 2020; Erickson, 2021). Sports vision training (SVT) aims to improve visuomotor abilities by increasing the usage intensity and frequency of the neural visual system, promoting physiological adaptations within the visual system (Appelbaum and Erickson, 2018; Poltavski et al., 2021; Vasile and Stănescu, 2024). Initially employed in medical rehabilitation, vision training has gained increasing attention in competitive sports as research deepens, becoming a significant field within sports science research (David et al., 2021; Erickson, 2021; Stein et al., 2024). Currently, SVT not only encompasses a broader spectrum of sports (Appelbaum et al., 2016; Forni et al., 2022; Hülsdünker et al., 2019; Vasile and Stănescu, 2024) but also explores in-depth the specific characteristics of various visual capabilities (David et al., 2021; Jurkiewicz et al., 2024; Schwab and Memmert, 2012; Wilkins and Appelbaum, 2020).

Visuomotor skills, which can be divided into different sub-ability, such as static and dynamic visual acuity, contrast sensitivity, eye-hand coordination (Buscemi et al., 2024; Erickson et al., 2011; Millard et al., 2023), play a crucial role in athletic performance. According to the visual information processing model proposed by (Welford, 1960), which athletes can gather visual information during competition and control the muscular system. The response can be devided into three consecutive stages: Perception, Decision, and Effector (Erickson, 2021). The perception mechanism involves receiving external information from sensory receptors such as visual, vestibular, auditory, and tactile inputs. This information is then passed to the decision mechanism, which not only stimulates response but also exhibits inhibitory control over actions in the face of adverse information. The preferred action plan is then transmitted to the effector mechanism, where the brain’s dorsal system organize, initiate, and control the generation of neural commands to stimulate the muscular system to act (David et al., 2021; Erickson, 2021). As the perception mechanism continuously receives ongoing visual information with including movements coordinated from previously issued commands. Therefore, the decision mechanism processes both the external feedback received by the perception mechanism and the internal feedback from the effector mechanism’s control of motion, thus achieving control and adjustment of response actions. For example, in some interception sports, athletes are required to have rapid visual processing abilities (Bonato et al., 2020; Causer et al., 2011).

Skeet shooting is an Olympic sport that heavily relies on visuomotor abilities, where shooters must observe and capture the high-speed flight of clay targets, quickly completing a series of actions such as mounting, moving, firing, and following through. The high initial speed (65–70 km/h), small size (110 mm) and rapid release (0–3 s after calling) of the targets pose significant challenges to the shooters’ visual tracking, target capture, and eye-hand coordination abilities. In this event, visuomotor abilities directly impact the shooters’ reaction time and accuracy. Excellent visuomotor skills enable shooters to quickly identify and respond to targets, accurately predict the trajectory, and maintain calm and focused attention under high-pressure conditions, effectively handling the complexity and variability of the competition. Athletes with rapid visuomotor response speed can process visual information more efficiently, making the flying clay target appear as if it were in “slow motion” (Nascimento et al., 2020). In similiar sports involving interceptive actions, Balasaheb et al. (2008) found that 6-week of SVT could improve cricket batters’ reaction time, depth perception, accommodation abilities, eye movement characteristics, and batting performance (*p* < 0.001), with no such changes in the control group. Liu et al. (2020) observed significant improvements in baseball players’ pitching angles and hitting distances after 8.5 hours of dynamic visual training, suggesting that such training can enhance precision in controlling objects and improve both launching and interceptive actions. Furthermore, numerous studies (Appelbaum et al., 2016; Ellison et al., 2020; Joseph et al., 2020; Millard et al., 2023; Poltavski et al., 2021; Schwab and Memmert, 2012; Stephen et al., 2013) have also demonstrated that SVT can enhance athletic performance in various other sports.

Given the critical impact of visuomotor abilities on athletic performance and active exploration of various training methods by coaches and researchers to enhance this capability (Appelbaum et al., 2016; Buscemi et al., 2024; Laby and Appelbaum, 2021), this study focuses on the improvement of visuomotor abilities in skeet shooters. The goal is to systematically assess and identify specific deficiencies in athletes’ visual abilities and improve their specialized shooting ability through SVT, thereby helping athletes enhance their performance.

## 2 Methods

### 2.1 Participants

Twenty elite skeet shooters were recruited for the training, data collection and randomly divided into an experimental group (EXP, *n* = 10) and a control group (CON, *n* = 10). All participants had previously competed in international competitions and finished in the top three. Age, experience and gender presented in Table 1 for example. Before the training intervention, participants completed relevant questionnaires to confirm the absence of migraines, epilepsy, other neurological disorders, or medication usage through a questionnaire, eliminating risks associated with SVT. None of the participants had undergone similar SVT before. This study was approved by the Shanghai University of Sport Research Ethics Committee (No. 102772023RT182) and performed in accordance with the Helsinki Declaration. Informed consent was obtained for all participants included in this study.

**TABLE 1:** Basic information of participants.

Group	Age (yrs)	Sex (male/ female)	Training years (yrs)	Best record in qualification (pcs)
EXP	25.1 ± 5.4	5 / 5	10.3 ± 4.4	121.4 ± 2.5
CON	25.0 ± 4.1	5 / 5	10.4 ± 3.8	121.3 ± 2.1

### 2.2 Experimental protocols

The experimental design of this study consisted of a pre-test, 6-week of SVT, and a post-test, conducted from December 2023 to January 2024. Pre- and post-test assessments included visuomotor skills testing, hit accuracy testing, shotgun-mounting reaction time testing, and gun barrel kinematics testing. The hit accuracy were based on participants’ scores from two major competitions—the National Championship (end of November 2023) and the National Team Monthtly Trail (mid-January 2024). The other three tests were completed one day before and after the intervention. SVT was conducted under stable indoor lighting conditions that did not affect shooters’ visual capabilities, with each training session and evaluation test conducted by specially trained researchers. The 6-week SVT included two sessions per week, totaling 12 sessions, with a total weekly intervention time not exceeding two hours. SVT performed using the Senaptec Sensory Station(Senaptec Inc, USA). The training programmes were determined based on previous studies (Appelbaum et al., 2016; Dendy et al., 2023; Shekar et al., 2021) and expert interviews with coaches and researchers in the field, specifying warm-up activation, training content, intervention duration, and rest intervals (Table 2). The relationship between the training items and specialization is explained in Table 3. The CON followed target tracking training in skeet shooting, enhancing their visuomotor abilities and gun control from a perspective closer to actual competition conditions. Besides the training content, the duration of each training session, rest intervals, and total training duration were controlled to be consistent between EXP and CON. Before and after training, the RPE subjective fatigue scale and a visual fatigue scale (Rajabi-Vardanjani et al., 2014) were used to monitor the physical load on shooters, ensuring equivalent intensity levels for both groups. Both groups continued to participate in their teams’ regular technical, physical, and psychological training outside the experiment to eliminate other training impacts on the visuomotor skills test results.

**TABLE 2:** Sports vision training programs.

Training items	Sets	Completion time	Rest interval
Warm-up activation (Reaction stick toss and catch)		5 min	
Eye-hand coordination	5	1 min	30 s
Go/No go	4	1 min	30 s
Dynamic vision	4	1 min	30 s
Perception training	4	1 min	30 s
Near far shift	4	30 s	30 s
Response inhibition	4	30 s	30 s
The total time	60min		

**TABLE 3:** Explanation to the relevance of training items.

Training items	Explanation of the specific relevance
Warm-up activation (Reaction stick toss and catch)	In skeet shooting, athletes require proficient eye-hand coordination, reaction speed, and upper limb flexibility to maintain stable shooting postures and movements. The reaction stick toss, an adaptation from tennis ball toss practices, serves as a common pre-competition activation technique. It necessitates athletes to accurately discern the color, trajectory, and speed of the airborne reaction stick, enabling rapid decision-making and hand movement adjustments. Additionally, color recognition enhances the shooters’ cognitive processing speed and attentional focus.
Eye-hand coordination	The flight time of a clay target after launch is extremely brief, demanding immediate responses from shooters. This requires not only high central visual acuity but also the use of peripheral vision to detect visual stimuli from the clay targets. The brain must make rapid decisions to coordinate bodily movements for shooting. Effective eye-hand coordination can reduce response times and maintain good body stability, allowing shooters to shoot at the optimal point during the target’s flight.
Choice reaction	During competitions, the variability of the shooting range, clay targets, and environment demands excellent decision-making skills from shooters. For instance, if broken targets or external objects enter their field of view, athletes must quickly cease their shooting response. This reduces the time spent on processing and judging disruptive visual information, preventing a”domino effect“that could impair subsequent shots.
Dynamic Vision	In skeet competitions, clay targets are launched at high initial speeds and are thrown within 0–3 s after the call. This requires shooters to quickly lock onto and judge their next actions. Dynamic visual training enhances shooters’ ability to capture and track targets in rapidly changing environments, improving the accuracy of prediction and decision-making. Through training, shooters can react more swiftly and process visual information more efficiently, enhancing their accuracy in capturing and shooting the clay targets.
Recognition speed	Recognition speed training evaluates whether skeet shooters possess the ability to memorize the clay targets’ stations and trajectories during competitions and to recreate these visual informations in their minds. The flight paths of clay targets in skeet are relatively stable, allowing shooters to observe and predict the trajectory based on the shooting process of the previous athlete, which aids in making informed decisions for subsequent shots. Additionally, during double-target shooting, shooters must quickly lock onto one target and complete the shot, then promptly form a visual image, memorize the initial trajectory, and search for the second target within a broader peripheral field of view to complete the shooting sequence.
Near far shift	In the training and competition of skeet shooting, shooters need to quickly adapt to the continuously changing distances of clay target flights and swiftly acquire visual information from various distances. This demands rapid focus adjustment abilities. Therefore, the capacity for quick and precise focus shifting plays an indispensable role in gathering essential visual information.
Response inhibition	During competitions, shooters must make rapid decisions to shoot or not based on the conditions on the field, within a very short timeframe. Hitting a broken target can psychologically burden a shooter, affecting their performance on subsequent shots. Successful anticipation can significantly boost a shooter’s confidence. Training in quick decision-making helps shooters make accurate judgments within tight deadlines, improving their hit rates.

### 2.3 Testing measures

#### 2.3.1 Visuomotor skills testing

Shooters were tested for visuomotor skills using the assessment modules of the Senaptec Sensory Station, including four tasks that measure perceptual abilities (Visual Clarity, Contrast Sensitivity, Near-Far Quickness, Target Capture), three that assess decision abilities (Depth Perception, Perception Span, Multiple Object Tracking), and three that evaluate response abilities (Reaction Time, Eye-hand Coordination, Go/No Go). Visual Clarity (VC) task assessed a minimum detectable spatial resolution. Contrast Sensitivity (CS) measures the ability to detect differences in luminance between an object and its background, crucial for seeing in low-light conditions. Near-Far Quickness (N/F Q) assesses the how quickly and accurately participants could switch focus between near and distant objects. Target Capture (TC) evaluates the ability to accurately and quickly locate and focus on a specific target within a visual field. Depth Perception (DP) assessed the smallest amount of disparity required to resolve differences in depth. Perception Span (PS) evaluates the amount of visual information a person can process and remember at one time. Multiple Object Tracking (MOT) assesses the ability to simultaneously track and monitor multiple moving objects in a dynamic environment. Reaction Time (RT) evaluates how quickly a person can respond to visual stimuli. Eye-hand Coordination (EHC) measures the synchronization of visual input with hand movements. Go/No Go (G/N G) tested the ability to rapidly respond to “go” targets while inhibiting responses to “no-go” non-targets. The indicators VC, CS, TC, and DS reflect the athletes’ visual sensitivity thresholds, while the remaining six indicators are used to evaluate athletes’ visual-motor abilities (Erickson, 2021; Gökşen and Ýnce, 2024; Wang et al., 2015). This equipment has been widely used to build models of sports vision abilities for different types of athletes and has been validated for reliability and effectiveness (Appelbaum et al., 2016; Wang et al., 2015). Detailed descriptions of these indicators and their testing methods are available in studies by Erickson et al. (2011) and Poltavski and Biberdorf (2015), with operational procedures and specific explanations presented in Table 4. According to Kristina et al. (2016), results for N/F Q, PS, EHC, and G/N G are influenced by the testers’ familiarity with the equipment and cognitive aspects, hence athletes are familiarized with the testing process and operations before the pre-test and post-test, and the principles and precautions of each test are explained to ensure reliable and valid data. Pre-tests and post-tests are conducted within the same day period, with instructions provided before each test to clarify specific requirements and operations. The testing concludes once an individual completes all items without breaks, with each test session lasting 20–25 min.

**TABLE 4:** Senaptec visuomotor skills test requirements and explanation of indicator types.

Indicator classification	Testing indicator	Operation mode	Data type (Unit)	Specific explanation
Perception skills	Visual Clarity (VC)	Measure static visual acuity of athletes. The participant holds a mobile phone while standing 3 meters away from a tablet. During the test, a black Landolt ring with a gap appearing randomly at the top, bottom, left, or right will be displayed on the tablet. The athlete must determine the direction of the gap and swipe in the corresponding direction on the mobile device. If the gap direction is difficult to discern, participants are allowed and encouraged to make educated guesses.	Mean Binocular Acuity (log MBA)	Logarithmic function of the size of the Landolt ring correctly judged by participants
	Contrast sensitivity (CS)	Test the visual system’s ability to distinguish objects from their surroundings under varying brightness conditions. Participants hold a mobile phone while standing on a line 3 meters away from a tablet. During the test, four black rings will appear on the screen, one of which will randomly contain a concentric circle with ripples. The participant must identify this concentric circle and swipe in the corresponding direction on the mobile device. If the direction of the gap is difficult to discern, participants are encouraged to make educated guesses.	Visual sensitivity at 6 cpd spatial frequency (log CS_6)	Logarithmic function of the percentage of sensitivity correctly judged by participants
	Near-far quickness (N/F Q)	Test the ability of the visual convergence function to adjust during movement, measuring the speed at which the focus of vision switches between distant and near objects. The participant holds a mobile phone with one arm extended, ensuring both the tablet and the phone are within the central visual field, 3 meters away from the tablet. During the test, Landolt rings alternately appear on the tablet and the mobile phone. The participant must determine the direction of the gap in the rings and quickly swipe in the corresponding direction on the phone. If the answer is incorrect, the target will not switch, with the test duration being 30 seconds.	Number of correct taps (score)	Number of Landolt rings correctly identified within 30 seconds
			Near-end switching time (ms)	Average time to switch gaze from tablet to mobile phone
			Far-end switching time (ms)	Average time to switch gaze from mobile phone to tablet
	Target capture (TC)	Test athletes’ jumping tracking abilities during visual-motor processes. The participant holds a mobile phone while standing 3 meters away from a tablet, keeping their gaze level with the center of the large screen. During the test, a Landolt ring will randomly appear at one of the four corners of the large screen. The athlete must determine the direction of the gap and swipe in the corresponding direction on the mobile device. The duration of the Landolt ring display will gradually decrease following a correct response.	Exposure time (ms)	Minimum exposure time on the large screen for Landolt rings with correctly judged gap direction by participants
Decision skills	Depth Perception (DP)	Reflect the athlete’s ability to focus on the spatial distance and station of objects. The participant holds a mobile phone while standing on a 3-meter line from a tablet and wears a pair of 3D glasses. During the test, four black circles will appear on the screen, one of which will exhibit a three-dimensional visual effect. The participant must identify this circle and swipe in the corresponding direction on the mobile device. If the direction of the gap is difficult to discern, participants are encouraged to make educated guesses.	Disparity threshold (arc sec)	Secant function of the angle difference correctly judged by participant
	Perception span (PS)	Assess the breadth of visual perceptual range and spatial memory capacity. The participant stands 60 cm away from a tablet with their eyes level with the screen center. During the test, a number of circles will radiate outward from the center of the screen. Some circles will briefly display a black dot at their centers for 100 ms. After the black dot disappears, the participant must identify and click on the circles where the black dots appeared.	Testing score (score)	Number of correct responses minus incorrect and unresponded attempts
	Multiple Object tracking (MOT)	Test the ability to simultaneously track multiple moving targets. The participant stands 60 cm away from a tablet with their eyes level with the screen center. During the test, pairs of black spheres will appear on the screen. In each pair, one sphere will briefly turn red before reverting to black. Subsequently, the spheres within each pair begin to rotate randomly in clockwise and counterclockwise directions. After several rotations, the movement stops, and the participant must identify the sphere in each pair that initially turned red.	Achieved level (level)	Number of correct circle matches completed, up to level 6
			Rotational speed (°/s)	The maximum rotation speed of the correct circle pair judgment.
Effector skills	Reaction Time (RT)	Test the response time of the dominant and non-dominant hands to simple stimulus signals. The participant stands 60 cm away from a tablet, with eyes level with the screen center. The dominant hand is determined at the start of the test. During the test, participants lightly touch the centers of two circular targets on the tablet with the pads of their index fingers, ensuring no other part of the hand touches the screen. Once aligned, the circles will turn blue. Randomly, one of the circles will turn red, prompting the participant to quickly lift and place down their finger in response. Both hands must not leave the screen simultaneously.	Bilateral hand response time (ms)	Average response time for both hands to stimuli
			Dominant hands (ms)	Average response time for dominant hand to stimuli
			Non-dominant hands (ms)	Average response time for non-dominant hand to stimuli
	Eye-hand Coordination (EHC)	Reflects the coordination between the visual system and the motor execution system, where the brain makes decisions based on visual information acquired and guides the body to execute commands quickly and accurately. The participant stands with their feet 60 cm away from a large screen, ensuring that both hands can touch the entire screen. During the test, among 80 circles arranged in 10 rows and 8 columns on the screen, a blue ball will randomly appear. The participant must react by touching the ball with either hand. Once a ball is touched, another will appear at a random location. A total of 80 balls will appear randomly during the test.	Average response time (ms)	Average time from target appearance to completion of clicks
			Central response time (ms)	Average time from appearance to click completion for targets in the central area
			Peripheral response time (ms)	Average time from appearance to click completion for targets in the peripheral area
			Distance from the circle center on the X-axis (mm)	Distance from the click location to the center of the blue ball on the X-axis
			Distance from the circle center on the Y-axis (mm)	Distance from the click location to the center of the blue ball on the Y -axis
	Go/No Go (G/NG)	On the basis of quickly and accurately locating targets, this test evaluates the participant’s ability to control their response inhibition and cognitive flexibility. Similar to the EHC test, during the test, blue and red balls will randomly appear on the screen and disappear within 450ms to jump to the next one. Participants need to click on the blue balls before they disappear while avoiding touching the red balls. During the test, 96 balls will appear (64 blue and 32 red).	Testing score (score)	Number of correct clicks minus incorrect and unresponded attempts

#### 2.3.2 Hit accuracy

The number of hits in shooting competitions is the most direct indicator of skeet shooters’ athletic performance (Causer et al., 2011). As the athletes recruited for this study are elite skeet shooters, shooting errors are less likely at station 1, 2, 6, 7, and 8. Station 3, 4, and 5, being further from the hit station, are defined as more challenging, and the finals of skeet shooting competitions also take place at these three stations, where hitting is more difficult (Bender, 2016). Top skeet shooters perform better at these stations, distinguishing the most skilled of elite group, hence station 3, 4, and 5 were chosen for shot hit count data collection. The qualification rounds consist of 5 rounds of shooting: station 3 (1 single target, 1 double target), station 4 (2 single targets, 2 double targets), and station 5 (1 single target, 1 double target), totaling 12 shots per shooter, with each shooter’s hits from 60 shots recorded during the qualification.

#### 2.3.3 Gun barrel kinematics

**FIGURE 1:**
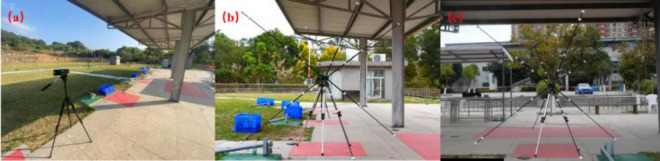
**(a)** Camera station diagram **(b)**: The perspective of Camera 1; **(c)** The perspective of Camera 2.

**FIGURE 2:**
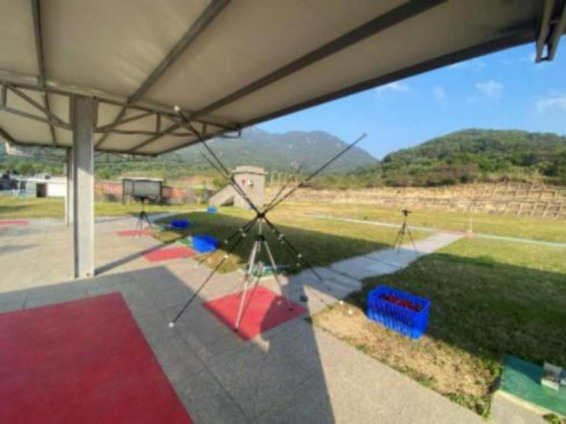
3D framework placement diagram.

Data collection for gun barrel kinematics follows the method mentioned by Causer et al. (2010). Three high-speed sports cameras (AX700; SONY, Japan) are used, sampling at a rate of 100Hz with a shutter speed of 1/1000. The three cameras are stationed 5.0 meters directly in front, to the left, and to the right of the shooting stations, forming a ‘T’ shape, set at a height of 1.05 meters (Figure 1). The cameras record simultaneously during each shooting trial. A three-dimensional framework with 28 points is used for calibration on the shooting station (Figure 2). Videos are analyzed using Simi Motion 9.2 software. Causer et al. (2010) found that elite skeet shooters perform better than non-elite shooters in three kinematic indicators: Peak velocity for shot 2, Displacement of gun shot 2 (horizontal axis), and Displacement of gun shot 2 (vertical axis), with significant differences between hits and misses, hence these three indicators are used to evaluate the impact of SVT on specific technical actions of skeet shooters. During testing, shooters wear professional shooting attire and goggles, using their training and competition shotguns, shooting at station 4, sequentially shooting two double targets left and right, demanding all hits; if a miss occurs, the shot is retaken.

#### 2.3.4 Shotgun-mounting reaction time

**FIGURE 3:**
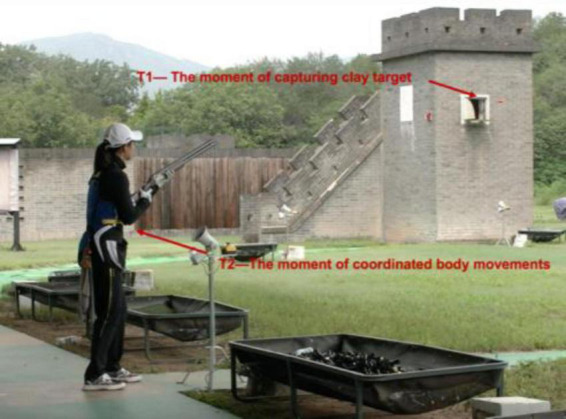
Camera shooting method of Shotgun-mounting Reaction Time (ΔT = T_2_–T_1_).

Sampling was performed using a high-speed sports camera (AX700; SONY, Japan), with a sampling rate of 100Hz and a shutter speed of 1/1000. The shooting requirements must capture both the target exit and the full body of the athlete. The moment the clay target exits the trap is recorded as T_1_; immediately after capturing the target’s flight, the athlete makes a decision through neural pathways, and the coherent initiation of the shotgun-mounting action is recorded as T_2_. The shotgun-mounting reaction time (ΔT = T_2_–T_1_) is calculated based on the difference between these two time points (Figure 3), used to evaluate the shooter’s ability to respond quickly from capturing visual information to directing the limbs. Dartfish 10.0 is used to analyze the videos, with testing requirements and data collection as mentioned above for the kinematic indicators.

### 2.4 Statistical analysis

Data collected are statistically analyzed and organized using IBM SPSS 27.0, with results presented as mean ± standard deviation (M ± SD). All test data undergo normality testing (*n* ≤ 50, using Shapiro-Wilk test) and homogeneity of variance testing (one-way ANOVA). For EXP and CON data before and after the intervention, horizontal analyses are conducted; data that meet normal distribution and pass homogeneity tests are analyzed using independent samples *T*-tests, otherwise, Mann-Whitney U-tests are used; for longitudinal analyses before and after the intervention, data meeting normal distribution and homogeneity are analyzed using paired samples *T*-tests, otherwise using Wilcoxon Signed-Rank tests. Test results with *p* < 0.05 indicate significant differences, and *p* < 0.01 indicate very significant differences.

## 3 Results

In the EXP, pre- and post-test comparisons show significant improvements in the number of correct taps in Near-Far Quickness, near-end switching time, and far-end switching time (*p* < 0.05). Scores in Perception Span, average response time in Eye-hand Coordination, and distances from the touch location to the circle center on the X and Y axes all showed very significant improvements (*p* < 0.01). The remaining 12 indicators did not show significant changes. The CON pre- and post-test comparisons showed no statistical differences (*p* > 0.05). Results of the visuomotor skills tests are shown in Table 5.

**TABLE 5:** The effect of SVT on visuomotor skills.

Testing indicator	Data type (Unit)	Testing time	EXP	CON	*t/Z*	*p*
Visual Clarity	Mean binocular acuity (log MBA)	Pre	−0.85 ± 0.23	−0.23 ± 0.14	−1.601	0.123
		Post	−0.15 ± 0.08	−0.18 ± 0.12	−0.336	0.796
Contrast Sensitivity	Visual sensitivity at 6 cpd spatial frequency (log CS_6)	Pre	2.14 ± 0.13	2.13 ± 0.12	1.695	0.107
		Post	2.16 ± 0.08	1.99 ± 0.38	−1.214	0.353
Near-Far Quickness	Number of correct taps (score)	Pre	28.3 ± 8.0	26.9 ± 10.0	0.989	0.349
		Post	36.6 ± 4.5^#^	23.1 ± 6.1	5.642	0.000**
	Near-End switching time (ms)	Pre	801.67 ± 144.97	849.18 ± 165.39	−0.151	0.733
		Post	674.21 ± 80.34^#^	918.83 ± 348.91	−2.344	0.019*
	Far-End switching time (ms)	Pre	1093.09 ± 353.30	1197.37 ± 516.04	−0.605	0.503
		Post	809.13 ± 62.76^#^	1285.21 ± 471.36	−3.403	0.000**
Target Capture	Exposure time (ms)	Pre	190.0 ± 44.4	187.5 ± 27.0	−0.992	0.353
		Post	160.0 ± 39.4	195 ± 38.7	−2.135	0.035*
Depth Perception	Disparity threshold (arc sec)	Pre	98.5 ± 90.6	157.0 ± 71.2	−1.72	0.089
		Post	67.3 ± 69.3	102.7 ± 76.5	−1.485	0.143
Perception Span	Testing score (score)	Pre	39.2 ± 20.9	40.7 ± 10.6	−0.568	0.579
		Post	54 ± 7.6^##^	42.5 ± 7.1	3.502	0.003**
Multiple Object Tracking	Achieved level (level)	Pre	4.8 ± 0.8	5.0 ± 0.8	−0.564	0.631
		Post	5.0 ± 0.8	5.1 ± 0.6	−0.294	0.796
	Completion time (ms)	Pre	512.6 ± 114.9	481.2 ± 166.5	−0.265	0.796
		Post	501.6 ± 142.2	509.6 ± 140.6	−0.127	0.901
Reaction Time	Both hand response time (ms)	Pre	310.0 ± 26.2	311 ± 26.2	−0.026	0.980
		Post	309.0 ± 27.9	315 ± 34.9	−0.396	0.697
	Dominant hands (ms)	Pre	314.1 ± 29.8	319.4 ± 35.9	−0.359	0.723
		Post	313.5 ± 25.8	317.3 ± 33.1	−0.286	0.778
	Non-dominant hands (ms)	Pre	306.6 ± 27.0	302.0 ± 30.3	0.358	0.724
		Post	306.2 ± 33.1	312.6 ± 39.2	−0.394	0.698
Eye-hand Coordination	Average response time (ms)	Pre	551.14 ± 48.15	563.64 ± 27.08	−0.907	0.393
		Post	511.41 ± 28.95^##^	555.41 ± 37.52	−2.936	0.009**
	Central response time (ms)	Pre	503.87 ± 57.97	502.08 ± 28.14	−0.529	0.631
		Post	473.48 ± 34.74	497.22 ± 31.96	−0.921	0.369
	Peripheral response time (ms)	Pre	559.02 ± 57.56	590.03 ± 33.23	0.655	0.529
		Post	542.67 ± 29.49	580.35 ± 43.34	−1.670	0.036*
	Distance from the circle center on the X-axis (mm)	Pre	12.05 ± 1.43	12.27 ± 1.27	−0.605	0.579
		Post	10.80 ± 0.86^##^	11.68 ± 1.52	−1.593	0.129
	Distance from the circle center on the Y-axis (mm)	Pre	12.04 ± 1.44	12.28 ± 1.23	−0.529	0.631
		Post	10.76 ± 0.88^##^	11.69 ± 1.49	−1.701	0.106
Go/No Go	Testing score (score)	Pre	14.9 ± 7.4	13.5 ± 7.4	0.425	0.676
		Post	20.1 ± 3.7	12.7 ± 4.9	−3.073	0.002**

* indicates a significant difference between the test data of two groups of athletes at *p* < 0.05;

** indicates a highly significant difference at *p* < 0.01;

^#^ indicates a significant difference between pre-test and post-test at *p* < 0.05;

^##^ indicates a highly significant difference between pre-test and *post-test* at *p* < 0.01.

Post-intervention results in the EXP compared to the CON showed significant differences in near-end switching time in Near-Far Quickness, exposure time in Target Capture, and peripheral target response time in Eye-hand Coordination (*p* < 0.05). There were highly significant differences in the number of correct taps in Near-Far Quickness, far-end switching time, scores in Perception Span, average response time in Eye-hand Coordination, and scores in Go/No Go (*p* < 0.01). The other indicators showed no statistical differences (*P* > 0.05).

According to the results shown in Table 6, after 6 weeks of SVT, shooters in the EXP showed a very significant improvement in hit accuracy (*p* < 0.01), with similarly significant differences compared to the CON. The CON did not show improvement in hit numbers at these three stations (*p* > 0.05).

**TABLE 6:** The effect of SVT on hit accuracy.

Testing indicator	Data type (Unit)	Testing time	EXP	CON	*t*	*p*
Hit Accuracy	Hit Accuracy at Station 3, 4, 5 (pcs)	Pre	50.8 ± 2.2	50.6 ± 1.4	0.241	0.812
		Post	54.3 ± 2.3^##^	51.0 ± 2.0	3.414	0.003**

** indicates a highly significant difference at *p* < 0.01;

^##^ indicates a highly significant difference between pre-test and post-test at *p* < 0.01.

Subsequent specialized technical tests on shooters in the EXP (Table 7) revealed that the shotgun-mounting reaction time showed a highly significant improvement (*p* < 0.001). In the three kinematic indicators of gun barrel movement during the shooting process of double targets, peak velocity (*p* < 0.001), displacement on the X-axis (*p* = 0.033), and displacement on the Y-axis (*p* = 0.001) also showed significant improvements.

**TABLE 7:** The effect of SVT on shooting performance.

Testing indicator	Data type (Unit)	Pre	Post	*t*	*p*
Gun Barrel Kinematics	Peak velocity for shot 2 (m/s)	1.322 ± 0.062	1.294 ± 0.057	−3.989	0.000^##^
	Displacement of gun shot 2-horizontal axis (cm)	22.619 ± 4.172	21.919 ± 3.947	2.205	0.033^#^
	Displacement of gun shot 2-vertical axis (cm)	3.058 ± 1.084	2.684 ± 1.061	3.539	0.001^##^
Shotgun-mounting Reaction Time	Reaction Time (s)	0.189 ± 0.025	0.177 ± 0.029	−4.052	0.000^##^

^#^ indicates a significant difference between pre-test and post-test at *p* < 0.05;

^##^ indicates a highly significant difference between pre-test and post-test at *p* < 0.01.

## 4 Discussion

Skeet shooting, unlike rifle and pistol disciplines, requires shooters to intercept dynamic targets from static positions (Causer et al., 2010). This demands acute visual processing to predict trajectories and control shotguns effectively by coordinating internal and external feedback. SVT enhances visuomotor skills, translating to improved performance in specific tasks. While SVT appears to bolster some cognitive skills in skeet shooting, its broader impacts and underlying mechanisms merit further investigation.

### 4.1 The effect of SVT on visuomotor skills

During competitions, skeet shooters need to see “accurately” rather than “clearly”. Surveys of shooters have revealed a high incidence of myopia, including severe cases, among these athletes. Furthermore, no studies have found that SVT can enhance VC. Popescu et al. (1969) found that good CS is necessary for skeet shooters to differentiate fast-moving targets from the background. A statistical analysis of visual capability indicators for 2317 athletes in the Senaptec database showed that the top 5% of CS scores were about 2.00. Our results corroborate these findings, with both EXP and CON achieving CS scores around 2.00, reflecting elite performance. Brown et al. (2021) further demonstrate that champion skeet shooters, compared to regular soldiers, exhibit enhanced stereoscopic and color vision, being more sensitive to light and color. Notably, skeet shooters utilize specialized sports glasses that protect their eyes and enhance visual perception by adapting to various lighting conditions and improving color contrast during competitions. Consequently, these athletes generally exhibit superior CS that does not necessitate further targeted enhancement. The most notable perceptual improvement observed post-SVT was in N-F Q, where both near- and far- switching time were significantly reduced, enabling shooters to lock onto targets faster and track them more efficiently. Despite inconclusive pre and post-test results for TC within the EXP, significant differences were noted between EXP and CON. TC test is very similar to the skeet shooters’ preparation process, where the shooter focuses on the shotgun hold-point and perceives the target emerging from the window through peripheral vision. Lower time indicate better focus and capture abilities, and a quicker response to zero-delay target targets (Burris et al., 2020). Unlike multi-participant sports (Hülsdünker et al., 2019) with varied external influences, skeet shooting involves fewer variables, placing a greater emphasis on the shooters’ neural decision and response systems to guide muscular actions rather than external visual stimulus processing.

Decision ability allows athletes to select optimal actions during competition (Forsman et al., 2016). High-level athletes possess superior decision-making speed (Klatt et al., 2021; Zheng et al., 2024) and are capable of choosing the best option from a multitude of information, along with having exceptional visual search abilities (Bruce et al., 2012; Natsuhara et al., 2020; Ribeiro et al., 2021). While previous studies often focus on team (Moeinirad et al., 2020; Ribeiro et al., 2021) or net-barrier sports (Cardoso et al., 2021) requiring comprehensive field awareness, skeet shooting also demands rapid decision-making due to the predictable trajectory of clay targets and minimal external disturbances. Of the three tested indicators, only PS showed significant improvement, crucial for swiftly and accurately tracking targets (Erickson et al., 2011) (Burris et al., 2020). Higher PS abilities in shooters can help them quickly and accurately track flying targets and shorten the time from detection to shooting response. DP, the ability to analyze three-dimensional environments (Presta et al., 2021). Elite athletes perform better in integrating visual and visuomotor skills to control interception actions, demonstrating their precision in judging the spatial distance and relative speed between themselves and targets (Gao et al., 2015). DP is difficult to significantly improve through SVT, as even athletes with lower DP levels only achieve moderate improvements with specific visual training (Mazyn et al., 2007). The complexity of skeet shooting, often involving simultaneous targeting, highlights the critical ability, Multiple Object Tracking, of shooters where maintaining attention on double targets is challenging. Achieving higher MOT scores, such as the highest level 6 in the Senaptec test, may require extended SVT, as evident by the consistent level 5 scores in both EXP and control CON, suggesting the potential benefits of prolonged training.

The most significant effects of SVT in this study were observed in response abilities. RT showed no improvement for either the dominant or non-dominant hand. This outcome aligns with previous findings that the top 5% of athletes exhibit RTs above 309.57 ms. Burris et al. (2020), indicating a robust ability among shooters to respond to simple stimuli. Furthermore, Henrique et al. (2021) suggest that even with extensive training, futsal players do not achieve notably faster reaction times than the general population, indicating a need for more specialized training to enhance this capability. Additionally, the uniform response time between hands, which can benefit overall motor coordination and speed (Badau et al., 2018), suggests that training plans for skeet shooters should focus on enhancing coordination balance between hands. EHC is a focal point of current research into athletes’ visuomotor abilities, emphasizing the synchronization of hand movements with visual stimuli (Bajaj et al., 2024; de Brouwer and Spering, 2022; Erickson, 2021; Jurkiewicz et al., 2024). Post-SVT, the EXP exhibited faster response speeds and greater accuracy, although improvements in response time for central and peripheral targets were not significant. Relative to target-tracking training, SVT also improved shooters’ response accuracy but not speed, showing less enhancement than expected. In fast-action sports like skeet shooting, superior EHC allows shooters more time to track and follow targets. Acute stroboscopic vision training significantly improved EHC, although this benefit diminishes after a washout period of 10 days (Ellison et al., 2020; Stephen et al., 2013). Contrarily, Appelbaum et al. (2016) observed that 11 weeks of sports vision training (SVT) did not substantially enhance college softball players’ eye-hand coordination (EHC). This lack of improvement is likely due to the lower training intensity compared to stroboscopic vision training. These findings underscore that enhancements in EHC are closely linked to the duration and intensity of SVT. The G/N G, assessing athletes’ peripheral eye-hand response time to visual stimuli, is distinct from the EHC test as it requires athletes to quickly respond to “Go” stimuli and minimize responses to “No Go” stimuli. This capability is essential for skeet shooters who need to quickly react to broken and irregularly moving targets—penalized if not signaled to the referee in time. Following 6-week of SVT, there were notable improvements in G/N G scores, reflecting significant advancements in Choice Reaction and Response Inhibition. This training effectively enhanced the shooters’ decision-response capabilities and alleviated the psychological stress associated with non-effective responses during competitions.

### 4.2 The effect of SVT on shooting performance

Previous studies have found that SVT can enhance athletes’ precision performance, such as the defensive success rates of elite badminton players (Hülsdünker et al., 2019), the penalty kick accuracy of soccer players (Giustino et al., 2024), the hitting accuracy of tennis players (Forni et al., 2022). A notable study by Causer et al. (2011) on quiet eye training for international-level skeet shooters demonstrated that the average duration of quiet eye increased from 397ms to 423ms, and the onset of the quiet eye phenomenon occurred earlier (from 257ms to 244ms), ultimately improving shooting accuracy from 62% to 70%. This earlier and longer focus helps elite shooters to perceive the visual stimuli of the targets more quickly and enter the decision phase, while a longer duration of quiet eye allows athletes to more accurately sense the direction, speed, and distance of the targets relative to the gun barrel, reducing the impact of stimulus-driven control and enabling the best response strategies. Our findings reveal that in the EXP, the number of successful hits at difficult stations increased by 5.83%, whereas the CON showed negligible change. This suggests that a combination of multiple visuomotor skills, enhanced through SVT, contributes to these improvements, warranting further exploration through detailed kinematic analysis during shooting sequences.

Reaction time, or premotor time, begins with the initial stimulus and ends when the response starts, followed by movement time, during which the limbs complete the action (Ando et al., 2001). This reaction time is influenced by skill level (Ando et al., 2001) and gender (Ak and Koçak, 2010). In skeet shooting, shotgun-mounting reaction time is critical—it is the duration from when a shooter perceives the target to when they decide to mount the shotgun, followed by the physical response. Our findings indicate a significant reduction in shotgun-mounting reaction times post-SVT in the EXP, likely due to improved EHC and enhanced G/N G response abilities. This quicker decision-making and response execution enable the hit area of the targets to fall closer to the center of the range, not only bring the hit location closer to the muzzle, reducing the impact of “trajectory drop”, but also reserve more time for tracking, following then firing the second shot for shooters. Additionally, improvements in TC testing time and PS facilitate quicker recognition of peripheral targets, crucial in the fast-paced environment of skeet shooting. This enhanced peripheral object recognition contributes to more precise and timely responses in competitive settings. Notably, the shotgun-mounting reaction time in specialized testing environments proved significantly shorter than the response time in RT, influenced by the slower nerve transmission speeds and distances from the central nervous system (Schmidt and Lee, 1982), and compensated by predictive abilities (Kesting and Treiber, 2006). Athletes familiar with their sporting environment often exhibit shorter reaction times, benefiting from anticipatory skills (Günay et al., 2019; Hülsdünker et al., 2021). However, the lack of significant change in reaction time among the EXP before and after SVT suggests that further research is needed to understand if SVT can enhance athletes’ ability to compensate for slow reactions with prediction.

Improvements in visuomotor abilities significantly impact skeet shooters not only during the shotgun-mounting phase but throughout the entire shooting process. Causer et al. (2010) observed that top skeet shooters exhibit reduced gun barrel displacement on the horizontal axis during the shot 2 and achieve lower peak velocity when tracking the second target. Further studies by Causer et al. (2011) through 8-week quiet eye training significantly reduced these metrics, demonstrating that decreased gun movement and minimal reverse acceleration contribute to more stable shooting. Our findings similarly show that six weeks of SVT enhance these key performance indicators. After firing the first shot, shooters must swiftly align with the next target, requiring increased gun barrel acceleration to match the target’s speed. During this critical phase, the visual nerve system actively coordinates with the rapid movement of the target, significantly aided by N/F Q and EHC abilities. Reductions in vertical (Y-axis) gun barrel displacement indicate improved stability in shitgun control. The recoil force from the ammunition causes significant muzzle bounce after the first shot is fired, which would cause the gun barrel to point too high relative to the trajectory of the flying target. A smaller Y-axis muzzle displacement indicates that the shooter adjusts the muzzle to a more appropriate height before reversing the gun, achieving the ideal station relative to the airborne target more quickly, thus reducing the peak speed and X-axis displacement of the muzzle movement. Enhancements in EHC precision also bolster shooters’ rapid targeting abilities, transferring skills honed on static targets to dynamic scenarios in competitive environments. Compared to the improvement in precision in EHC tests among CON shooters, SVT can achieve better training benefits, and even improve more visuomotor abilities and shooting performance. As an advanced visual training method, SVT significantly surpasses traditional target-tracking training.

### 4.3 Limitation and future directions

Despite achieving some expected experimental results, this study has some limitations that need to be addressed in future research: (1) All participants were elite athletes already performing at very high levels on multiple indicators, making it difficult to fully observe the enhancement effects of SVT on these high-level indicators, which may affect the external validity of the study results. (2) Due to competition scheduling, the training intervention lasted only six weeks. Although some indicators improved, they did not reach statistically significant differences. (3) While this study shows that SVT enhances the athletic performance of skeet shooters, it has not yet detailed the relative effects of variables such as training intensity, intervention duration, and training content. Different intensities and types of SVT may have varying impacts on athletic performance, and future research should systematically assess the independent and interactive effects of these factors.

## 5 Conclusion

This study found that a 6-week SVT intervention can effectively improve the visuomotor abilities of skeet shooters, including Near Far Quickness, Target Capture, Perception Span, Eye-hand Coordination, and Go/No Go. By integrating this training with specialized shooting technique training, it enhanced their hit accuracy and precision of shotgun control, providing them with shorter shotgun-mounting reaction time and more economical control accuracy of shot 2 action, optimizing their overall shooting performance. This provides a strong scientific basis and practical support for future training in skeet shooting.

## Data Availability

The datasets presented in this study can be found in online repositories. The names of the repository/repositories and accession number(s) can be found in the article/Supplementary material.
